# Current developments and opportunities of pluripotent stem cells-based therapies for salivary gland hypofunction

**DOI:** 10.3389/fcell.2024.1346996

**Published:** 2024-01-19

**Authors:** Wenpeng Song, Huan Liu, Yingying Su, Qian Zhao, Xiaoyan Wang, Pengfei Cheng, Hao Wang

**Affiliations:** ^1^ Department of Stomatology, Beijing Tiantan Hospital, Capital Medical University, Beijing, China; ^2^ Beijing Laboratory of Oral Health, School of Basic Medicine, School of Stomatology, Capital Medical University, Beijing, China; ^3^ Research and Development Department, Allife Medicine Inc., Beijing, China; ^4^ Biochemistry and Molecular Biology, School of Basic Medical Sciences, Beijing, China; ^5^ Department of Stomatology, Eye Hospital, China Academy of Chinese Medical Sciences, Beijing, China

**Keywords:** pluripotent stem cells, induced pluripotent stem cells, salivary gland hypofunction, salivary gland, regenerative therapy, immunoregulation

## Abstract

Salivary gland hypofunction (SGH) caused by systemic disease, drugs, aging, and radiotherapy for head and neck cancer can cause dry mouth, which increases the risk of disorders such as periodontitis, taste disorders, pain and burning sensations in the mouth, dental caries, and dramatically reduces the quality of life of patients. To date, the treatment of SGH is still aimed at relieving patients’ clinical symptoms and improving their quality of life, and is not able to repair and regenerate the damaged salivary glands. Pluripotent stem cells (PSCs), including embryonic stem cells (ESCs), induced pluripotent stem cells (iPSCs), and extended pluripotent stem cells (EPSCs), are an emerging source of cellular therapies that are capable of unlimited proliferation and differentiation into cells of all three germ layers. In recent years, the immunomodulatory and tissue regenerative effects of PSCs, their derived cells, and paracrine products of these cells have received increasing attention and have demonstrated promising therapeutic effects in some preclinical studies targeting SGH. This review outlined the etiologies and available treatments for SGH. The existing efficacy and potential role of PSCs, their derived cells and paracrine products of these cells for SGH are summarized, with a focus on PSC-derived salivary gland stem/progenitor cells (SGS/PCs) and PSC-derived mesenchymal stem cells (MSCs). In this Review, we provide a conceptual outline of our current understanding of PSCs-based therapy and its importance in SGH treatment, which may inform and serve the design of future studies.

## 1 Introduction

**FIGURE 1:**
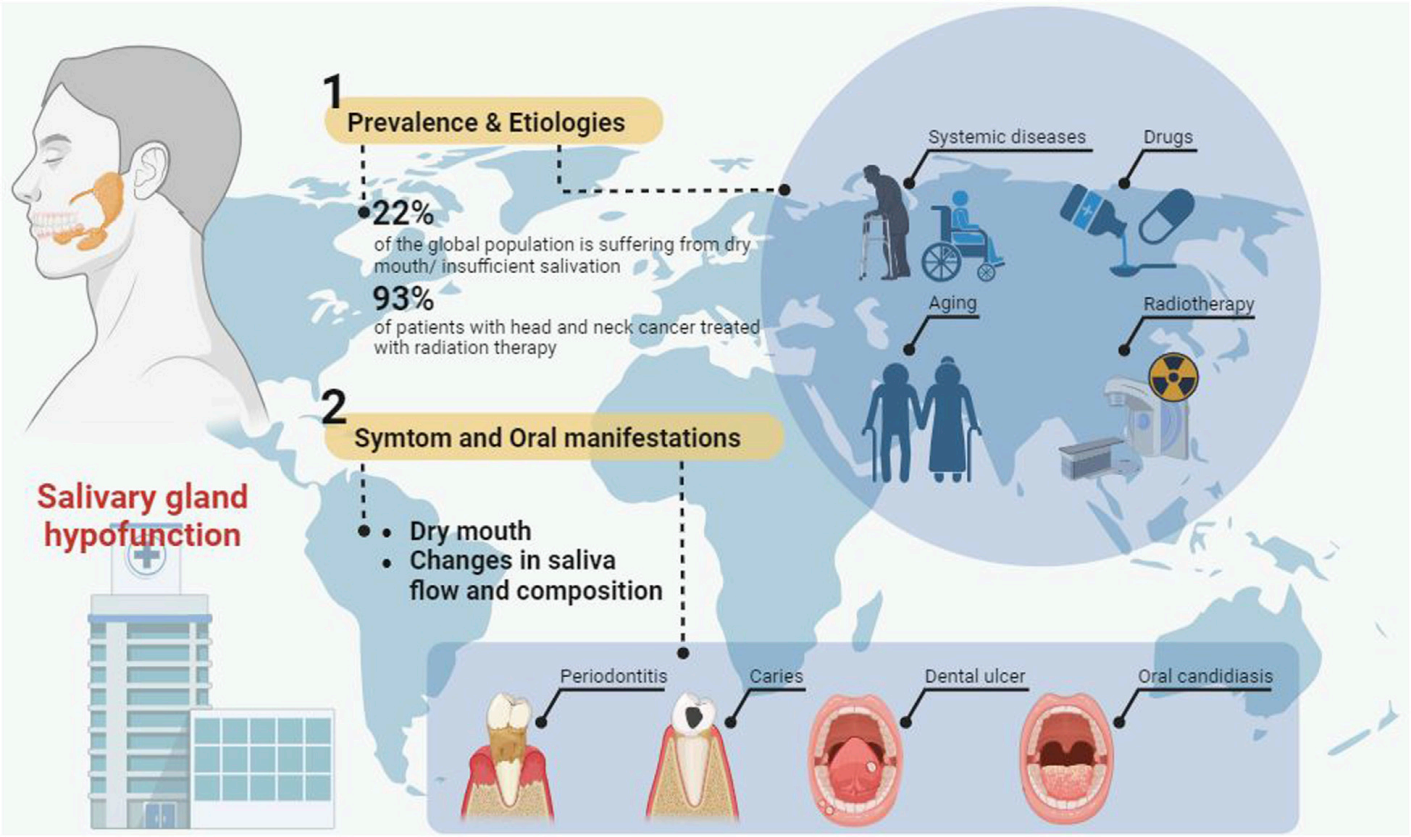
The prevalence, etiology, symptoms, oral manifestations and treatment of salivary gland hypofunction.

Saliva assists in maintaining oral and general health. On the one hand saliva has buffering and remineralizing properties and plays a crucial role in digestion, taste, cleaning, moistening the mouth and protecting the teeth (Saleh et al., 2015). On the other hand, the antibacterial, antifungal and antiviral properties of saliva can maintain the balance of the oral microbiota and protect the body from harmful external factors (Salum et al., 2018). Salivary gland hypofunction (SGH) is a common oral health condition caused by a variety of etiologies, including systemic diseases, drugs, aging, and radiotherapy for head and neck cancer (Figure 1). Salivary glands hypofunction leads to dry mouth, decreased salivary flow and altered salivary composition, which can increase the risk of oral ulcers, dental caries, gingivitis, periodontitis, oral candidiasis, salivary gland infections (Nederfors, 2000; Saleh et al., 2015). Oral health conditions such as periodontitis and dental caries may also contribute to the development of a variety of systemic diseases, including cardiovascular and metabolic diseases (Figure 1) (John et al., 2016; González Navarro et al., 2017). It has been reported that up to 22% of the global population is suffering from dry mouth (Agostini et al., 2018). At present, the main treatment strategies for SGH are symptomatic treatment (e.g., saliva substitutes) and salivary stimulants (e.g., pilocarpine and cevimeline) (Villa et al., 2015). However, due to the lack of repair and regeneration of the damaged salivary gland tissue, these treatments do not provide satisfactory therapeutic effects (Villa et al., 2015).

In recent years, a number of cell therapy strategies based on salivary gland stem/progenitor cells (SGS/PCs) and mesenchymal stem cells (MSCs) have been reported as promising solutions to the current therapeutic dilemma of SGH. In 2012, the study by Xu et al. (2012) indicated that intravenous infusion of allogeneic umbilical cord MSCs (UCMSCs) was able to increase oral salivary flow in patients with Sjogren’s syndrome (SS). Two clinical studies by Lynggaard et al. (Lynggaard et al., 2022a; Lynggaard et al., 2022b) also found that intraglandular allogeneic adipose tissue-derived mesenchymal stem/stromal cells (AT-MSCs) injections alleviated the symptoms of radiation-induced xerostomia. Additionally, in multiple preclinical studies based on animal models of SGH, intraglandular injection of salivary gland progenitor cells (SGPCs) rescued radiation-induced hyposalivation (Lombaert et al., 2008; Nanduri et al., 2013; Xiao et al., 2014; Pringle et al., 2016). However, the invasive operation for cell isolation, the limited number of isolated cells, the insufficient cell expansion capacity and the heterogeneity of cells have limited the wide application of adult cells in clinical therapy (Zhou et al., 2021).

Pluripotent stem cells (PSCs) with unlimited proliferative capacity and potential for the differentiation into three germ layers and their derived cells are expected to replace adult cells and become a potential important source in the treatment of SGH. PSCs can be derived from three kinds of cells, including embryonic stem cells (ESCs), induced pluripotent stem cells (iPSCs), and the newly discovered extended pluripotent stem cells (EPSCs) (Ohnuki and Takahashi, 2015; Tan et al., 2021). From the therapeutic point of SHG, on the one hand, PSCs are capable of possessing salivary gland regenerative potential through differentiation into salivary gland cells and MSCs (Dupuis and Oltra, 2021; Zhang et al., 2023). On the other hand, PSC-derived MSCs are expected to alleviate SGH caused by inflammatory diseases or autoimmune diseases through immunomodulation (Hai et al., 2018; Hu et al., 2023). In addition to direct cell transplantation, paracrine products of PSCs and their derived cells (e.g., extracellular vesicles (EVs) and conditioned medium) have both immunomodulatory and tissue regeneration-promoting potentials (Harrell et al., 2019; Fujii and Miura, 2022). In 2018, Bo et al. (Hai et al., 2018) revealed that iMSCs and iMSCs-derived EVs diminished lymphocyte infiltration in the salivary glands and decreased serum autoantibody levels in NOD mice (animal model of SS). In another study, EVs derived from young, rather than aged, iMSCs were found to inhibit the progression of SS by promoting M2 macrophage polarization and reducing Th17 cells in the spleen (Zhao et al., 2023). Additionally, transfection of a miR-125b inhibitor into aged iMSCs restored the biological activity of their derived EVs (Zhao et al., 2023).

Currently, the clinical application of PSCs is still partially limited, including their tumorigenicity, immunogenicity, and heterogeneity (Yamanaka, 2020). In addition, ESCs and their derived cells are limited by ethical and policy issues, making it difficult to apply to clinical treatment (Halevy and Urbach, 2014; Kolagar et al., 2020). The utilization of iPSCs/EPSCs, their derived cells, or paracrine products as therapeutics is expected to address these issues. Meanwhile, due to the similarity between different types of PSCs, studies based on the treatment of ESCs are still instructive for iPSCs and EPSCs.

Considering these aspects of information, PSCs may play a prominent role in the future treatment of SGH. This review began by describing the etiology of SGH and existing treatment options to highlight the need to develop new therapeutic strategies for SGH. Current knowledge and application potential of PSCs as a novel therapeutic strategy for SGH were also outlined in the following.

## 2 Overview of salivary glands

The anatomical structure, histology, and function of salivary glands have been extensively reviewed in multiple research (Holmberg and Hoffman, 2014; Patel and Hoffman, 2014; Aure et al., 2019; Chibly et al., 2022). The salivary glands are exocrine organs responsible for the production and secretion of saliva. They mainly include the parotid gland, submandibular gland, sublingual gland, and the small salivary glands distributed throughout the oral cavity (Chibly et al., 2022). Recently, Valstar and colleagues (Valstar et al., 2021) reported a pair of additional salivary glands in the nasopharynx near the torus tubarius, named tubarial glands. Mammalian salivary glands are branched organs formed by complex ductal trees, ultimately giving rise to secretory units called acini. Acini consist of serous acinar cells and mucous acinar cells. The saliva produced by acinar cells is modified by the ductal system, including intercalated ducts, striated ducts, and excretory ducts, and transported to the oral cavity (Valstar et al., 2021). Intercalated ducts are formed by a single layer of cuboidal cells containing small secretory granules with lysozyme and lactoferrin (Chibly et al., 2022). Striated ducts participate in the bidirectional transport and reabsorption of electrolytes and contain small secretory granules with the secretion of kallikreins and glycoproteins (Tandler and Phillips, 2000; Tandler et al., 2001). Excretory ducts are formed by tall columnar pseudostratified epithelium and are responsible for the reabsorption of sodium ions and the secretion of potassium ions, contributing to the production of final low-osmolarity saliva (Catalán et al., 2009). Muscle epithelial cells surround acinar and ductal cells, and they contract in response to neuronal stimulation to promote saliva secretion (Zyrianova et al., 2019).

The major salivary glands are innervated by both the sympathetic and parasympathetic nervous systems. Generally, the submandibular gland is responsible for the production of most unstimulated saliva, while the salivary secretion of the parotid gland is mainly responsive to stimulation. The parasympathetic nervous system stimulates the secretion of serous saliva through acetylcholine and substance P, while the sympathetic nervous system responds to norepinephrine, regulating mucous secretion, peripheral blood flow, and inflammation (Chibly et al., 2022). In addition, an increase in calcium ions in the cytoplasm is necessary for activating ion channels and transport proteins, thus generating the osmotic gradient required for saliva secretion (Kasai and Augustine, 1990).

Drug-induced or disease-related organic damage to the salivary glands or disruptions in neural pathways can lead to the occurrence of dry mouth. For example, some anticholinergic drugs may block the binding of acetylcholine to muscarinic receptors in the salivary glands (Arany et al., 2021). When certain diseases occur, such as Sjogren’s syndrome, ductal cells and acinar cells of the salivary glands may be damaged, thereby affecting saliva secretion (Jonsson et al., 2011).

The establishment of various animal models with SGH enables the study of salivary gland damage, repair, potential mechanisms, and the assessment of the effectiveness of treatment methods, including models involving duct ligation, radiation injury, mechanical injury (Kim et al., 2020). Extensive research based on animal experiments has found that the proliferation and transdifferentiation of salivary gland cells play a crucial role in maintaining homeostasis and repairing damage in the salivary glands (Aure et al., 2015; Ninche et al., 2020; Shubin et al., 2020; Chibly et al., 2022). For example, the study by Weng and colleagues (Weng et al., 2018) found that both acinar cells and ductal cells of the salivary glands are involved in the regeneration of acinar cells following radiation injury, indicating the involvement of cellular plasticity in salivary gland repair. In duct ligation injury models, muscle epithelial cells and ductal cells regenerate the damaged acinar cells (Ninche et al., 2020). The use of live cells to restore, enhance, or replace salivary gland function is showing promising results. On one hand, isolated or induced salivary gland cells labeled with salivary gland stem/progenitor cell markers are expected to treat salivary gland diseases by directly replacing damaged salivary gland cells or forming organoids to substitute for salivary gland tissues (Lombaert et al., 2008; Chibly et al., 2022). On the other hand, the regulatory immune response and tissue repair capabilities exhibited by adult MSCs/iMSCs and their paracrine products may also play a therapeutic role in treating autoimmune-induced SGH (Chibly et al., 2022).

## 3 The etiology of SGH

### 3.1 Systemic disease

Changes in the composition and flow rate of saliva may be associated with a variety of systemic diseases such as autoimmune, endocrine, neurologic/mental, infectious, and genetic disorders that result in neurotransmitter receptor dysfunction, destruction of glandular parenchyma, or immune dysregulation (Table 1) (Saleh et al., 2015).

**TABLE 1:** Systemic diseases related to the occurrence and development of salivary gland hypofunction.

Classification of diseases	Diseases
Autoimmune disease	SS Kim et al. (2023)
	IgG4-related disease Li et al. (2015)
	Rheumatoid arthritis Aliko et al. (2010); Silvestre-Rangil et al. (2017)
	Juvenile idiopathic arthritis Kobus et al. (2017)
	Systemic lupus erythematosus Ben-Aryeh et al. (1993)
	Diffuse systemic scleroderma Parat et al. (2020)
	Primary biliary cirrhosis Mang et al. (1997)
Endocrine disease	Diabetes Moore et al. (2001), López-Pintor et al. (2016)
	Thyroid disorders Jung et al. (2017), Naik and Vassandacoumara, (2018)
Neurological/mental disorders	Alzheimer’s disease Ashton et al. (2019)
	Age-related cognitive decline Sørensen et al. (2018)
	Parkinson’s disease Verhoeff et al. (2023)
	Anxiety and depression Milin et al. (2016), Gholami et al. (2017)
	Burning mouth syndrome Poon et al. (2014), Spadari et al. (2015)
Communicable disease	COVID-19 Freni et al. (2020), Lechien et al. (2020), Omezli and Torul, (2021)
	AIDS Lin et al. (2003), Navazesh et al. (2003)
	Epstein-Barr virus Nakamura et al. (2020)
	Coxsackievirus Triantafyllopoulou et al. (2004)
	Human T-lymphotropic virus type 1 Nakamura et al. (2020)
	Hepatitis C virus Ramos-Casals et al. (2005)
	Hepatitis D virus Weller et al. (2016)
	*Helicobacter pylori* Wang et al. (2022)
Genetic disease	Salivary gland hypoplasia Pham Dang et al. (2010), Seymen et al. (2017)
	Ectodermal dysplasia Nordgarden et al. (2003)
	Prader-Willi syndrome Saeves et al. (2012a), Saeves et al. (2012b)
	Down syndrome Yarat et al. (1999), Areias et al. (2012), Domingues et al. (2017)
	Cystic fibrosis da Silva Modesto et al. (2015)
	Familial amyloid polyneuropathy Johansson et al. (1992)
	Fanconi anemia Mattioli et al. (2010)
	Gosher’s disease Dayan et al. (2003)
	Papillon-Lefèvre syndrome Lundgren et al. (1996)
	Hemochromatosis Rao et al. (2021)
	Mucopolysaccharides Nunes et al. (2022)
	Neurofibromatosis type 1 Cunha et al. (2015)
Other diseases	Hypertension Kawamoto et al. (2021), Ramírez et al. (2023)
	Sarcoidosis Mansour et al. (2013)

AIDS, Acquired immune deficiency syndrome; COVID-19, Coronavirus disease 2019; SS, Sjogren syndrome.

#### 3.1.1 Autoimmune disease

SS is one of the autoimmune diseases most associated with SGH, with a prevalence of about 60 patients/100,000 inhabitants, primarily affecting the exocrine glands and causing dry mouth and dry eyes (Qin et al., 2015; Brito-Zerón et al., 2016). Similar to most autoimmune diseases, the pathogenesis of SS is not completely understood. Various cells and cytokines are involved in the pathogenic process of SS, including salivary gland epithelial cells, T cells, B cells, dendritic cells, interferon (IFN), interleukins, tumor necrosis factor (TNF), and chemokines (Tian et al., 2021; Verstappen et al., 2021; Negrini et al., 2022). In addition, some signaling pathways are involved in the damage and fibrosis of salivary glands in patients with SS, including the JAK/STAT pathway activated by the binding of IFN to its receptor, Toll-like receptor (TLR) pathway, cGAS-STING signaling pathway, and TGF-β1/SMAD signaling pathway (Sisto et al., 2021; Tian et al., 2021; Zhu et al., 2023). Targeting these cells (such as B cells and T cells), cytokines (such as IL-6, IL-1 and TNF), and signaling pathways (such as JAK signaling pathway) may help regulate the immune response and improve SS (Seror et al., 2021; Zhan et al., 2023).

IgG4-related disease is a newly recognized fibroinflammatory disorder characterized by elevated serum IgG4 levels, which is usually accompanied by massive inflammatory infiltration, swelling, fibrosis of the major salivary glands (Li et al., 2015; Avincsal and Zen, 2017). It was reported that dry mouth could be observed in 30% of patients with IgG4-related disease (Puxeddu et al., 2018). In addition to these, several other autoimmune diseases have also been reported that may be associated with an increased risk of SGH, including rheumatoid arthritis (Aliko et al., 2010; Silvestre-Rangil et al., 2017), juvenile idiopathic arthritis (Kobus et al., 2017), systemic lupus erythematosus (Ben-Aryeh et al., 1993), diffuse systemic scleroderma (Parat et al., 2020), and primary biliary cirrhosis (Mang et al., 1997).

#### 3.1.2 Endocrine disease

Diabetes mellitus is one of the most common endocrine diseases and is expected to affect 693 million adults worldwide by 2045 (Cole and Florez, 2020). About 12.5%–53.5% of diabetic patients (both type I and type II diabetes) usually suffer from dry mouth and insufficient salivation (Moore et al., 2001; López-Pintor et al., 2016; Manjushree et al., 2022). This may be related to diabetic neuropathy the use of drugs for diabetes treatment, and salivary gland parenchymal injuries (including oxidative stress injury) (Chen et al., 2020; Genco and Borgnakke, 2020; Fouani et al., 2021). Furthermore, an increasing number of studies have reported changes in the expression of various proteins in the saliva and glands of diabetes patients, including salivary amylase, sodium-glucose cotransporter 1 (SGLT1), nitric oxide synthase and tetrahydrobiopterin protein (NOS-BH4), bone morphogenetic protein 7 (BMP7), common salivary protein 1 (CSP1), aquaporins (AQP), muscarinic receptors, and heat shock protein 60 (Hsp60) (Fouani et al., 2021).

Thyroid disorders are another common group of endocrine disorders that manifest as abnormal production of thyroid hormones, including hyperthyroidism, hypothyroidism, thyroiditis, and Hashimoto’s thyroiditis. More than half of dry mouth sufferers are living with thyroid disease (Jung et al., 2017). In addition, some common treatments for thyroid disease, such as radioiodine therapy and thyroid hormone replacement therapy, may also exacerbate dry mouth symptoms (Bergdahl and Bergdahl, 2001; Smidt et al., 2011; Hesselink et al., 2016).

#### 3.1.3 Neurological/mental disorders

Salivary gland function is controlled by the autonomic nervous system and regulated by the central nervous system (Proctor, 2016; Sørensen et al., 2018). When affected by neurodegenerative diseases, salivary gland function may be altered accordingly (Proctor, 2016; Sørensen et al., 2018). Studies have shown that patients with neurodegenerative diseases such as Alzheimer’s disease, age-related cognitive decline, and Parkinson’s disease suffer from lower salivary flow rates and a higher risk of dry mouth compared to healthy individuals (Sørensen et al., 2018; Zalewska et al., 2021; Verhoeff et al., 2023). Furthermore, Zalewska and colleagues (Zalewska et al., 2021) have also identified oxidative stress imbalance and downregulation of total proteins in the saliva of Alzheimer’s disease patients. Although some studies have reported salivation problems in people with Parkinson’s disease, it may be related to a lack of control of their masticatory muscles (Verhoeff et al., 2023). The impact of mental disorders on salivary gland function remains controversial. Although some studies have found that anxiety and depression reduce the salivary flow rate and increase the risk of dry mouth (Milin et al., 2016; Proctor, 2016; Gholami et al., 2017), this may be an effect of antidepressant drugs rather than the disorder (Hunter and Wilson, 1995; de Almeida Pdel et al., 2008).

Burning mouth syndrome (BMS) is a condition often associated with the development of mental disorders such as anxiety and depression (Abetz and Savage, 2009). Despite the lack of clinical signs of neuropathy, several more accurate diagnostic methods have suggested the involvement of the small nerve fibers, the trigeminal nerve, the brainstem, and the central nervous system in patients with BMS (Jääskeläinen, 2018). The results of several randomized controlled studies have shown reduced unstimulated salivary flow rate (USFR) and stimulated salivary flow rate (SSFR) in patients with BMS (Poon et al., 2014; Spadari et al., 2015), but multiple others did not find differences in USFR and SSFR between patients suffering from BMS and healthy individuals (Aitken-Saavedra et al., 2022).

#### 3.1.4 Communicable disease

As of August 2022, Severe acute respiratory syndrome coronavirus 2 (SARS-CoV-2) has caused more than 59 million Coronavirus disease 2019 (COVID-19) cases and more than 6 million COVID-19-related deaths, and has become the greatest global public health threat of the century (Yuan et al., 2023). SARS-CoV-2 proliferates actively in the salivary glands, which directly affects the salivary glands and triggers SGH (Xu et al., 2020; Atukorallaya and Ratnayake, 2021). This was demonstrated in several clinical studies of COVID-19 patients with ongoing infection or treatment completion (Freni et al., 2020; Lechien et al., 2020; Omezli and Torul, 2021). According to data reported by the Joint United Nations Program on HIV/AIDS (UNAIDS, 2023), as of 2022 approximately 39 million people are living with acquired immune deficiency syndrome (AIDS) worldwide. Studies have shown that patients with AIDS have decreased salivary flow in the parotid, submandibular and sublingual glands in both the early and late stages of infection (Lin et al., 2003; Navazesh et al., 2003; Meer, 2019). Development of benign lymphoepithelial lesions of the salivary glands (including epimyoepithelial islands and an extensive lymphoid infiltrate) caused by long-term infection with human immunodeficiency virus (HIV) may play an important role in AIDS-associated dry mouth or SGH (Shanti and Aziz, 2009; Nizamuddin et al., 2018). In addition, several other sources of infection have been shown to induce a similar disease manifestation as SS (Maslinska and Kostyra-Grabczak, 2022), such as Epstein-Barr virus (Nakamura et al., 2020), coxsackievirus (Triantafyllopoulou et al., 2004), human T-lymphotropic virus type 1 (Nakamura et al., 2020), hepatitis C virus (Ramos-Casals et al., 2005), hepatitis D virus (Weller et al., 2016), and *Helicobacter pylori* (Wang et al., 2022).

#### 3.1.5 Genetic disease

Patients with salivary gland hypoplasia and ectodermal dysplasia are often characterized by underdevelopment or complete absence of large and small salivary glands, which in turn directly affects the formation and secretion of saliva (Nordgarden et al., 2003; Pham Dang et al., 2010; Seymen et al., 2017). Prader-Willi syndrome is a complex multisystem genetic disorder characterized by hyperphagia, hypotonia, growth hormone deficiency, and growth retardation (Vasconcelos et al., 2022). Although low salivary flow is a consistent manifestation in patients with Prader-Willi syndrome, the total amount of protein in saliva is not altered (Saeves et al., 2012a; Saeves et al., 2012b).

Down syndrome is a condition caused by a chromosomal abnormality (the presence of a supernumerary chromosome 21), with common symptoms of intellectual backwardness, peculiar facial features, growth disorders, and multiple malformations. The results of several studies suggest that individuals with Down syndrome have significantly lower salivary flow than healthy individuals (Yarat et al., 1999; Domingues et al., 2017). Saliva flow in children with Down syndrome is 36% lower than in healthy sibling pairs (Areias et al., 2012). In addition to these, a number of other genetic disorders have been reported to be associated with SGH or insufficient salivation, including cystic fibrosis (da Silva Modesto et al., 2015), familial amyloid polyneuropathy (Johansson et al., 1992), Fanconi anemia (Mattioli et al., 2010), Gosher’s disease (Dayan et al., 2003), Papillon-Lefèvre syndrome (Lundgren et al., 1996), hemochromatosis (Rao et al., 2021), mucopolysaccharides (Nunes et al., 2022), and neurofibromatosis type 1 (Cunha et al., 2015).

#### 3.1.6 Other diseases

Dry mouth and SGH are also commonly associated with hypertension. The cross-sectional study by Ramírez et al. (2023) included 221 hypertensive patients, who were assessed for dry mouth by asking questions and the Xerostomia Inventory and had their USFR recorded. The results of the study found that 51.13% and 37.56% of hypertensive patients were accompanied by dry mouth and reduced USSF, respectively (Ramírez et al., 2023). In another epidemiologic study that included 1,933 hypertensive patients, dry mouth was present in about 8% of hypertensive patients, which may be related to the reductions in the number of salivary gland acinar cells, enhanced fatty infiltration, and arterial stenosis (Kawamoto et al., 2021). Sarcoidosis, a chronic multisystem inflammatory granulomatous disease with an unknown origin that affects the salivary glands, exhibits mean salivary flow rates and total salivary protein similar to those in patients with SS (Mansour et al., 2013; Drent et al., 2021).

### 3.2 Aging

Aging is a spontaneous and inevitable process that affects almost all living organisms and is manifested by degenerative changes in tissue and organ structure and the decline in function. Histologic analyses of salivary glands from deceased individuals of different ages revealed the response of salivary gland parenchyma to aging (Scott et al., 1987). On the one hand, aging leads to a decrease in the mean volume of salivary gland follicles (submandibular glands by about 30%, labial salivary glands by about 25%, and parotid glands by about 12%), and a gradual increase in fatty infiltration and fibrotic tissue (Toan and Ahn, 2021). On the other hand, dilatation of salivary gland ducts and ductal irregularities in the elderly is also associated with aging (Scott, 1977; Scott et al., 1987). In addition to histologic changes, aging may induce impaired signaling in the adrenergic and muscarinic cholinergic receptor systems of the salivary glands, which reduces salivary gland physiological functions and responses (Miyamoto et al., 1993; Roth, 1995; Toan et al., 2021).

A meta-analysis published in 2015 systematically analyzed 47 studies on salivary flow in young and older adults and found that SFR and USFR were significantly lower in the older than in the young for the overall, submandibular, and sublingual glands, but not for the parotid and minor salivary glands (Affoo et al., 2015). In addition, this study also found that the SFR and USFR of the sublingual gland, as well as the overall USFR, were significantly lower in older adults than in younger adults, regardless of drug use (Affoo et al., 2015). These results suggest that the aging process is associated with reduced salivary flow and that medication use in the elderly does not fully explain the link between them.

In addition, several studies have found differences in salivary composition between healthy older adults and younger adults, such as an increase in salivary concentrations of K^+^, Ca^2+^, phosphate, amylase, and IgA and a decrease in total salivary Na^+^, Ca^2+^, Mg^2+^, IgG, and IgA (Nagler and Hershkovich, 2005; Nassar et al., 2014).

### 3.3 Impact of treatment modalities

#### 3.3.1 Drugs

Medications are the most common cause of dry mouth (Mese and Matsuo, 2007). A large number of drugs can trigger SGH, most commonly those acting on the nervous, cardiovascular, genitourinary, musculoskeletal, respiratory, and digestive systems, which act with receptors on the central nervous system and/or at the neuroglandular junctions, including muscarinic, α- and β-adrenergic, and certain peptidergic receptors (Femiano et al., 2008; Villa et al., 2016; Einhorn et al., 2020). For example, some anticholinergic drugs, including amitriptyline, hyoscine butylbromide, chlorpromazine, oxybutynin, and others, may potentially block the binding of acetylcholine to muscarinic receptors in the salivary glands, thereby inhibiting saliva secretion (Arany et al., 2021). An *in vivo* study also found that muscarinic receptor antagonists can induce histological and ultrastructural changes in the salivary glands of rats, leading to atrophy of glandular tissue (Aboulhoda and Ali, 2018).

In addition, the cytotoxic effects of different drugs on acinar and ductal cells and the inhibition of ion transport pathways in acinar cells may also be the causative factors of SGH (Hattori and Wang, 2007; Jensen et al., 2008). Chemotherapy is a well-established treatment for tumors (Weingart et al., 2018). The cytotoxicity of chemotherapy drugs varies depending on the type of chemotherapy drug and the type of cells, such as specific phases of the cell cycle, structures or functions of cell membrane (Jensen et al., 2008; Weingart et al., 2018). Some chemotherapy drugs, such as cyclophosphamide, epirubicin, methotrexate, and 5-fluorouracil, may lead to damage and impaired function of acinar cells and ductal cells in salivary gland tissues (Jensen et al., 2008).

Unfortunately, most of the data in these studies originated from patients’ chief complaints of dry mouth rather than from clinical examination of salivary flow rate, which may have contributed to the bias in the final results (Saleh et al., 2015). In most cases, drug-induced SGH can be resolved by discontinuing and replacing related drugs, and combined with symptomatic treatment (Einhorn et al., 2020).

#### 3.3.2 Radiotherapy

Radiotherapy is a common treatment for head and neck tumors and hyperthyroidism, including conventional radiotherapy, three-dimensional conformal radiotherapy, intensity-modulated conformal radiotherapy, proton beam radiotherapy, and radioiodine therapy (Jensen et al., 2010; Kim et al., 2018; Wiersinga et al., 2023). The physiological location of the salivary glands is more superficial than that of most head and neck tumors, and the radiation used for treatment usually needs to penetrate the salivary glands in order to effectively act on the deeper tumor tissues, which inevitably adversely affects the function of the salivary glands. For patients with head and neck cancer treated with conventional radiation therapy, three-dimensional conformal radiation therapy, and intensity-modulated conformal radiation therapy, 93% were reported to present with xerostomia or salivary gland hypofunction at the time of treatment, and 73.6%–85.3% at the post-treatment period (Jensen et al., 2010; Rigert et al., 2022). Also, after 1–2 years of treatment of thyroid cancer with radioactive iodine, 33.6%–37.8% of the patients also developed dry mouth symptoms (Jensen et al., 2010).

Notably, a dramatic loss of salivary gland function occurs within a week of radiation treatment and continues to downregulate salivary flow rates to unmeasurable levels throughout the course of treatment (Jensen et al., 2019). Progressive, irreversible changes in the salivary gland may be accompanied by the second stage of salivary gland hypofunction in the months following the completion of treatment (Jensen et al., 2010; Jensen et al., 2019). In this process, high doses of radiation may induce, on the one hand, selective damage to the cytoplasmic membrane of salivary gland acinar cells at an early stage, interfering with aqueous secretion after muscarinic receptor stimulation, and on the other hand, causing salivary gland acinar cells death, lack of cellular renewal, and impairment of ductal function at a later period (Konings et al., 2005). In addition, damage to vascular structures (increased capillary permeability, loss of capillary beds and arteriovenous fibrosis, interstitial edema, and inflammatory infiltration) may also be involved in radiotherapy-associated SGH (Cotrim et al., 2007; Jensen et al., 2019).

## 4 The clinical treatment of SGH

### 4.1 Symptomatic therapy

To date, there is no complete clinical cure for SGH, and the main goal of current treatment is to relieve the clinical symptoms and improve quality of life. Symptomatic treatment for reduced salivary secretion in patients with SGH is currently the most commonly used clinical treatment (Tulek et al., 2021). Early in the onset of SGH, patients can keep their mouths moist by drinking more water, but it does not possess salivary properties such as promoting lubrication and antimicrobial. Saliva substitutes with lubricants, thickeners, binders, and moisturizers as the main ingredients have been developed to combat these problems (Hu et al., 2021; Tulek et al., 2021), but there is still no consensus on the most effective ingredients or products to alleviate dry mouth (Plemons et al., 2014; Brito-Zerón et al., 2019). Several studies have reported the effectiveness of saliva substitutes in the control of hyposalivation and dry mouth, even though the duration of the effect may be short and repeated use is required (Boonroung and Vadcharavivad, 2011; Femiano et al., 2011; Ameri et al., 2016; Dalodom et al., 2016; Assery, 2019).

Insufficient salivation can lead to an imbalance in the oral flora, which may cause adverse oral health consequences such as caries, oral candidiasis, and periodontitis (Łysik et al., 2019). In addition to relieving dry mouth, several saliva substitutes with/without antimicrobial ingredients have been shown to help reduce bacterial flora, modulate the oral microenvironment, and control periodontal disease progression (Montaldo et al., 2010; Gookizadeh et al., 2012; Niemirowicz-Laskowska et al., 2020; Lam-Ubol et al., 2021). In addition, symptomatic treatments such as commercially available oral moisturizers, sprays, and mouthwashes have been reported to be promising in improving dry mouth in patients with SGH (Rhodus and Bereuter, 2000; Nieuw Amerongen and Veerman, 2003; Jose et al., 2016).

### 4.2 Salivary stimulants

In patients with SGH who still retain some salivary gland function, stimulation of salivary secretion using medication or other means can improve their dry mouth (Tulek et al., 2021). Pilocarpine and cevimeline are muscarinic and parasympathomimetic agonists that have been approved by the U.S. Food and Drug Administration for the treatment of dry mouth caused primarily by SS or radiotherapy (Plemons et al., 2014). Five placebo randomized controlled trials evaluating the therapeutic effects of pilocarpine (three trials) and cevimeline (two trials) in patients with SS found that pilocarpine and cevimeline significantly improved dry mouth (improvement in dry mouth from 24% to 31% in the placebo group to 61%–70% in the pilocarpine-treated group; and from 35% to 37% in the placebo group to 66%–76% in the cevimeline-treated group) and increased salivary flow rate (Vivino et al., 1999; Fife et al., 2002; Petrone et al., 2002; Papas et al., 2004; Wu et al., 2006).

Bethanechol is another parasympathomimetic agonist that is resistant to hydrolysis by acetylcholinesterase with a long-duration of effect (Jham et al., 2007). In 2023, Moral Nakamura et al. (2023) conducted a meta-analysis of three clinical studies on the use of bethanechol for treating dry mouth induced by radiotherapy for head and neck cancer. The results showed that bethanechol increased SSFR (Std. MD 0.66, 95% CI 0.28–1.03, *p* < 0.001) after radiotherapy, and USFR during (Std. MD 0.4, 95% CI 0.04–0.76, *p* = 0.03) and after (Std. MD 0.45, 95% CI 0.04–0.86, *p* = 0.03) radiotherapy (Moral Nakamura et al., 2023). It has also been shown that bethanechol is effective in increasing salivary secretion in xerostomic denture wearers (Singh et al., 2020) and significantly reduces acute salivary gland injury caused by radioiodine therapy (Campanhã et al., 2022).

Nizatidine is a histamine H2 receptor antagonist that effectively inhibits the enzyme acetylcholinesterase, and was generally used in the past for the treatment of gastric ulcers and gastroesophageal reflux disease (Kounenis et al., 1988; Nin et al., 2008). A study by Nin et al. (2008) found that nizatidine significantly increased SSFR and USFR in patients with dry mouth and healthy individuals, and resulted in subjective improvement of oral dryness in 65.8% of patients. In another study, nizatidine significantly increased salivation in patients with SS, but this promoting effect was not seen after treatment with another histamine H2 receptor antagonist, famotidine (Kasama et al., 2008).

Through the separate or combined effects of chewing and taste, chewing gum has the potential to increase the flow rate of saliva (Kim et al., 2021; Dodds et al., 2023). A recent meta-analysis found that chewing gum had a significant overall effect on salivary flow outcomes (SMD = 0.44, 95% CI 0.22–0.66; *p* = 0.00008), was able to increase USSF in older adults and patients with xerostomia, and may be associated with improved their levels of self-reported dry mouth (Dodds et al., 2023). In addition, acupuncture and electrical stimulation have been reported as alternative treatments for dry mouth and SGH, promising to accelerate the salivary flow rate and improve salivary gland function (Assy and Brand, 2018; Paim É et al., 2019; Salimi et al., 2021).

### 4.3 Biologic response modifiers

Persistent SGH is one of the most common manifestations of SS. It is still controversial whether it is possible to modulate autoimmunity and thereby improve salivary gland function in patients with SS by applying biological response modifiers (Wang et al., 2023). Rituximab, an anti-CD20 monoclonal antibody, effectively depletes B cell lineage in patients with SS (Souza et al., 2016). A randomized placebo-controlled study by Meijer et al. (2010) found that rituximab could significantly improve USFR and SSFR in patients with SS and effectively improve their dry mouth. However, these results were not able to be replicated in the study by Devauchelle-Pensec et al. (2014). Besides improving USFR, Bowman et al. (2017) also did not find any clinical benefit of rituximab on SS-induced SGH.

Similarly, although some studies have reported the ameliorative effect of IFN-α on SGH in patients with SS (Shiozawa et al., 1998; Ship et al., 1999; Khurshudian, 2003), no significant differences in SSFR and oral dryness were found between the IFN-α group and the placebo group in the study by Cummins et al. (2003).

### 4.4 Stem cell therapy

Although conventional treatments, such as salivary stimulants and saliva substitutes, are able to alleviate the symptoms of SGH to some extent, their effectiveness still depends on the number of remaining functional salivary gland acini. When salivary glands have suffered extensive damage with low regenerative capacity, regenerative therapies based on multiple stem cells are expected to be more effective than conventional treatments by replacing and repairing damaged salivary gland acini cells or by directly regenerating salivary gland tissue (Phan et al., 2023). Furthermore, the immunomodulatory capacity of both MSCs and MSC-derived exosomes has been demonstrated in preclinical studies of inflammatory and autoimmune diseases (Xu et al., 2012; Shen et al., 2021). Modulation of immune cells and the immune microenvironment using stem cells and their paracrine products is expected to ameliorate SGH caused by autoimmune diseases.

To date, there are several clinical reports on the use of stem cells for treating SGH or dry mouth. In 2012, Xu et al. (2012) attempted to treat patients with SS using intravenous infusion of allogeneic UCMSCs. It was found that the USFR of 11 patients with dry mouth increased after 1 month of cell transplantation, while the SSFR of these patients also increased significantly after 2 weeks, 1 month, 3 months, and 6 months of cell transplantation. Lynggaard et al. (2022a) applied direct transplantation of allogeneic ADSCs into the submandibular and parotid glands to treat patients with dry mouth induced by radiotherapy for head and neck tumors and found that 4 months after cell transplantation, the USFR (increased from 0.13 mL/min to 0.18 mL/min) and SSFR (increased from 0.66 mL/min to 0.75 mL/min) increased significantly and dry mouth were relieved. Another of their studies used the direct injection of autologous ADSCs into the submandibular gland in patients with radiotherapy-induced SGH and xerostomia. Although no statistically significant upregulation of USSF and SSFR was observed in the cell transplantation group versus the healthy control group in this study, the alleviation of dry mouth reported by the patients suggests a clinical benefit of the treatment (Lynggaard et al., 2022b).

The results of animal studies on different causes of SGH also suggested the therapeutic potential of multiple stem cells, including bone marrow mesenchymal stem cells (BMSCs), dental pulp stem cells (DPSCs), and exfoliated deciduous tooth stem cells (SHEDs), which are expected to expand the application of stem cell therapy for SGH (Chihaby et al., 2021; Guan et al., 2023).

### 4.5 Prophylactic treatment

Radiotherapy may result in severe SGH, and prophylactic medication to promote survival and proliferation of acinar cells prior to radiotherapy is expected to substantially reduce salivary gland damage and preserve more salivary gland function. In 2017, a meta-analysis by Cochrane stated that low-quality evidence suggests that amifostine (an organic thiophosphate) reduces the risk of moderate to severe dry mouth at the end of (RR 0.35, 95% CI 0.19–0.67; *p* = 0.001) and 3 months after (RR 0.66, 95% CI 0.48–0.92; *p* = 0.01) radiotherapy, meanwhile, increase USSF up to 12 months after radiotherapy (Riley et al., 2017).

In addition to amifostine, there is still insufficient evidence to determine whether there is a beneficial effect of other interventions such as pilocarpine, palifermin, biperiden in combination with pilocarpine, bethanechol, artificial saliva, selenium, antimicrobial mouthwash, and antimicrobial lozenges on radiotherapy-induced SGH (Riley et al., 2017).

### 4.6 Gene therapy

In 2012, a clinical trial by Baum et al. (2012) transferred aquaporin-1 cDNA via adenovirus to the parotid glands of 11 radiotherapy-treated patients with head and neck cancer suffering from SGH, and observed an increase in salivary flow in some of the patients (6/11) after 42 days, with five of them also feeling relief from dry mouth.

Another study by the research group followed up 5 patients who responded to the previous treatment for 3–4 years and found that the parotid flow of all patients was significantly increased, suggesting that aquaporin-1 cDNA transfection has a positive long-term effect on parotid salivary secretion (Alevizos et al., 2017). It is worth noting that there are no high-quality reports on the use of gene therapy for SGH. Whether gene therapy can safely and effectively provide clinical benefits to patients with SGH is still unclear.

## 5 Potential role of PSCs in the treatment of SGH

### 5.1 Treatment by PSC-derived salivary gland cells

#### 5.1.1 Treatment by direct transplantation of PSC-derived salivary gland cells

**FIGURE 2:**
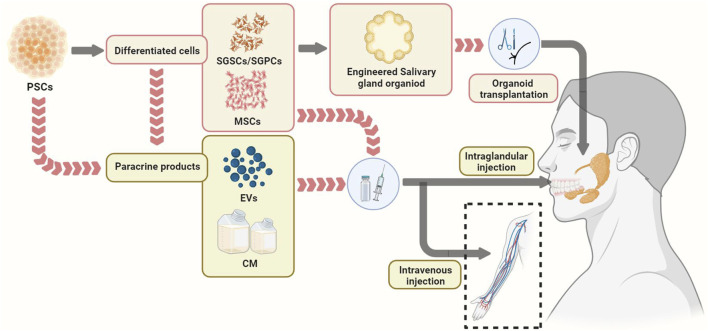
Potential pathways of PSCs in the treatment of salivary gland hypofunction, including cell transplantation, transplantation of paracrine products of cells, and transplantation of engineered salivary glands organoid. EVs, Extracellular vesicles. MSCs, mesenchymal stem cells, PSCs, pluripotent stem cells; SGPCs, salivary gland progenitor cells; SGSCs, salivary gland stem cells.

**FIGURE 3:**
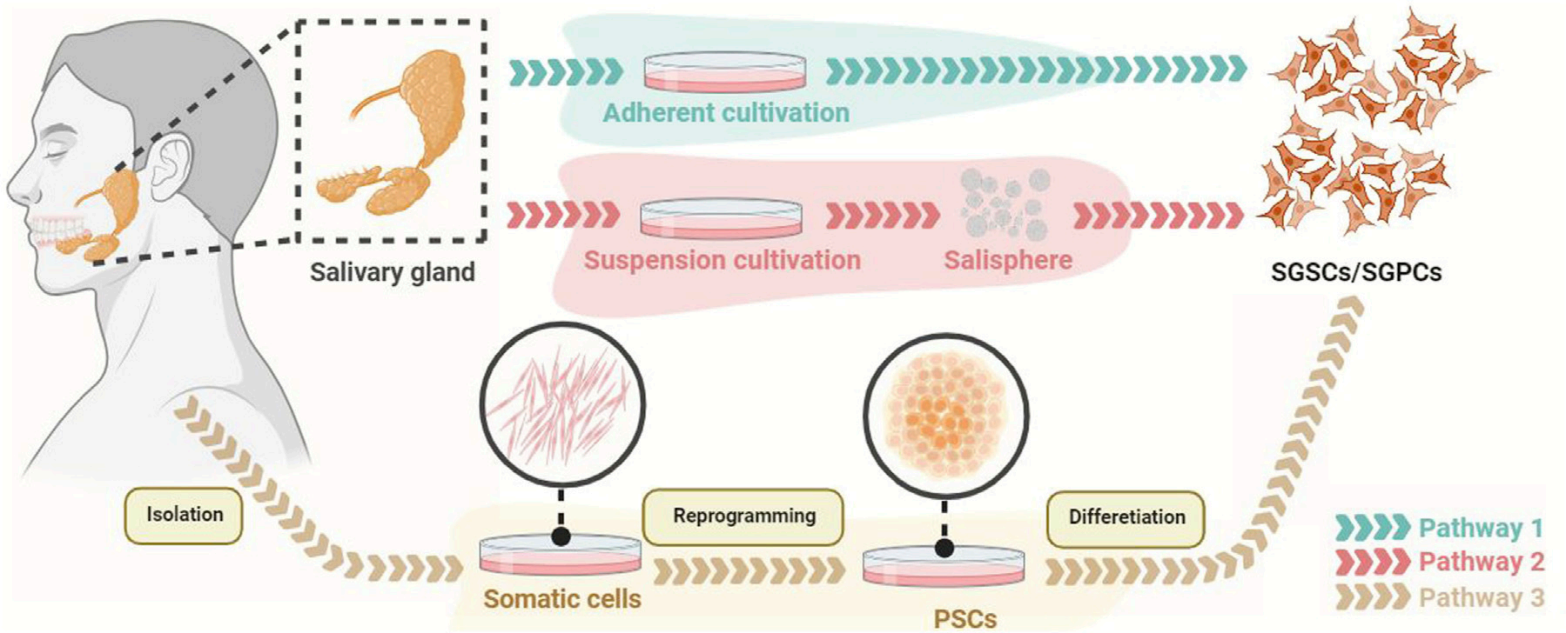
The methods for obtaining SGSCs/SGPCs. SGSCs/SGPCs are able to be obtained by adherent culture or suspension culture (generating salisphere). In addition, PSCs derived from somatic reprogramming can be differentiated into SGSCs/SGPCs. PSCs, pluripotent stem cells; SGPCs, salivary gland progenitor cells; SGSCs, salivary gland stem cells.

**FIGURE 4:**
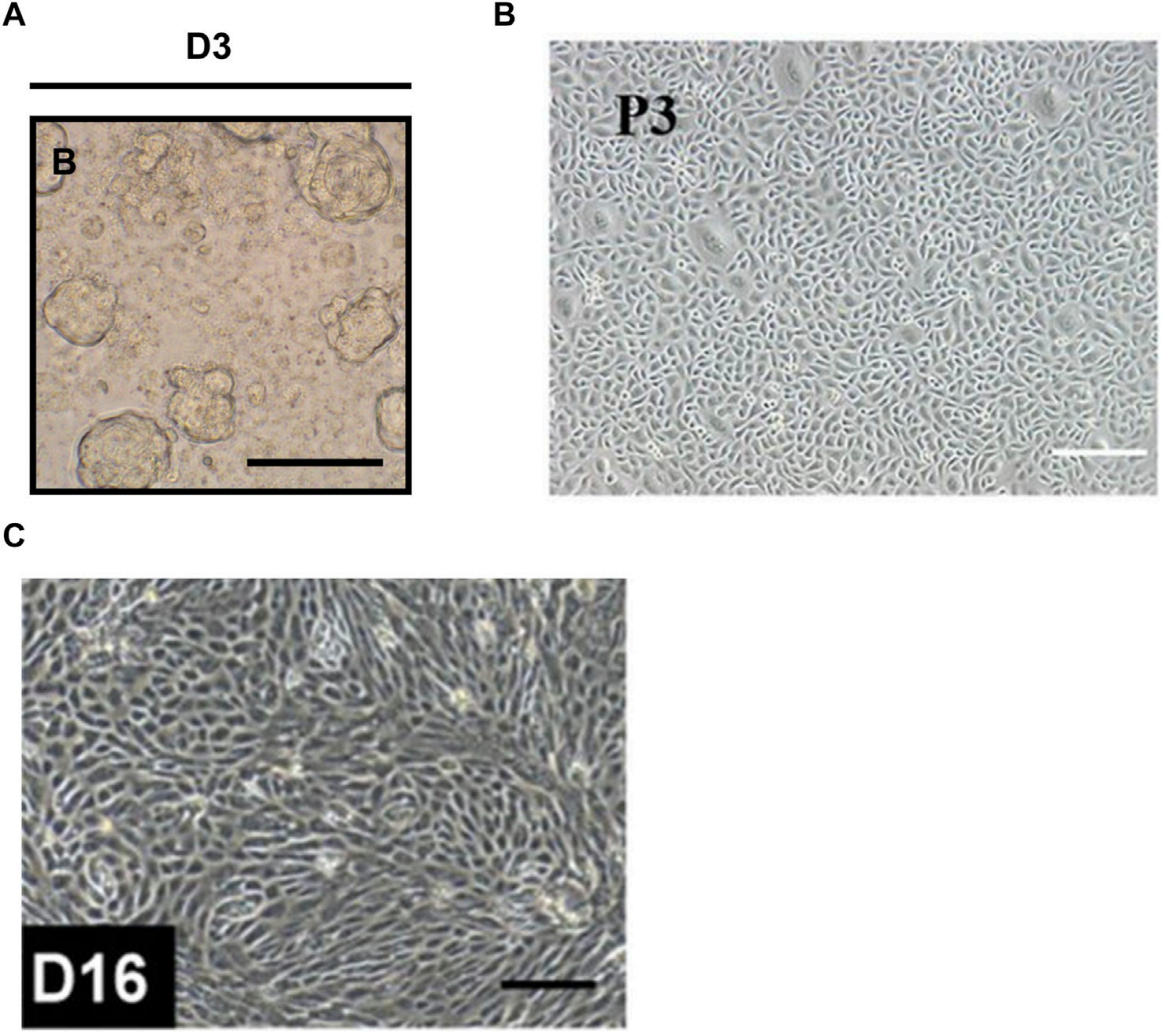
Brightfield images showed the morphology of the salivary gland progenitor cells. **(A)** The morphology of salisphere. Scale sec11bar = 50 µm. **(B)** Salivary gland progenitor cells isolated from human submandibular gland. Scale bar: 200 μm. **(C)** Human ESCs-derived salivary gland progenitor cells. Scale bar = 200 μm. The figures were adapted from reference (Lombaert et al., 2008; Sui et al., 2020; Zhang et al., 2023) with permission (Supplementary Figure S1).

Cell-based therapies aim to utilize living cells to restore, enhance or replace organ function (Chibly et al., 2022). Transplantation of PSC-derived cells, paracrine products, and engineered salivary gland organoids are several potential methods for PSCs in the treatment of SGH (Figure 2). SGSCs/SGPCs are the potential cell sources for treating salivary gland diseases, which can be obtained by *in vivo* isolation (by two-dimensional culture or suspension culture to form salispheres) and PSCs-induced differentiation (Lombaert et al., 2008; Sui et al., 2020; Chansaenroj et al., 2021; Zhang et al., 2023) (Figures 3, 4).

In 2008, Lombaert et al. (2008) successfully formed and maintained salispheres containing c-Kit^+^ SGPCs by suspension culture of mice submandibular gland cells (Figure 4A). In this study, intraglandular injection of only a few c-Kit^+^ cells (300–1,000 cells/gland) rescued salivary secretion in most mice (69%, 9/13) with radiation-induced salivary gland injury (Lombaert et al., 2008). In further studies, c-Kit^+^CD24^+^Sca1^+^ and c-Kit^+^CD24^+^CD49f^+^ mice SGSCs/SGPCs transplants also proved to be able to contribute to the restoration of salivary gland function and tissue homeostasis in mice with radiotherapy-induced SGH (Nanduri et al., 2013; Xiao et al., 2014). Notably, c-Kit^+^ SGPCs also exist in human salivary glands and can be isolated and cultured *in vitro* (Feng et al., 2009; Lombaert et al., 2013). A groundbreaking study using human salivary gland-derived c-Kit^+^ SGSCs/SGPCs successfully rescued radiotherapy-induced salivary insufficiency in the mice model (Pringle et al., 2016). This may be related to self-renewal and differentiation of transplanted SGSCs/SGPCs (Pringle et al., 2016).

With the increasing understanding of repairing salivary gland damage and SGSCs/SGPCs, it is becoming clear that SGSCs/SGPCs transplantation may have the potential to regenerate salivary glands. Although still controversial, in addition to c-Kit, proteins such as KRT5, KRT14, Axin2, SOX2, SOX9, SOX10, LGR5, CD90, and others have been recognized as markers of SGSCs/SGPCs (Yi et al., 2016; Chibly et al., 2022). Treating SGH by transplanting SGSCs/SGPCs with different markers is worthy of further exploration.

In addition to isolation from the salivary gland, SGSCs/SGPCs can also be obtained by PSCs differentiation (Figure 4C). In 2022, the protocol proposed by Zhang et al. (2023) successfully differentiated hESCs and hiPSCs into human salivary epithelial progenitor cells expressing SOX9, CD24, α-SMA, KRT5, and KRT19. In this scheme, ESCs and iPSCs need to be first formed into embryoid bodies (EBs) under suspension conditions, and then sequentially treated with BMP4, retinoic acid, and CHIR99021 in adherent culture, and ultimately differentiated into salivary epithelial progenitor cells with a similar transcriptome profile to that of human submandibular glands through the oral ectoderm (Zhang et al., 2023). In another of their studies, BMP4, retinoic acid, and FGF10 were used to promote ESC differentiation into salivary gland substrates *in vitro* (Zhang et al., 2022). Notably, in addition to the formation of invaginated epithelium and the development of initial budding, the expression of salivary gland progenitor cell markers such as KRT5, KRT19, SOX9, and E-cadherin was found in the ESC-derived salivary gland placodes, suggesting the differentiation ability of PSCs to salivary gland epithelial progenitor cells (Zhang et al., 2022).

In addition, the differentiation of PSCs into salivary gland cells, including acinar and ductal cells, also has the potential for therapeutic applications. In recent years, the induction of ESCs and iPSCs into salivary gland cells by co-culture with salivary gland cells, the use of salivary gland cell-conditioned medium, and direct salivary gland transplantation has been successively reported (Kawakami et al., 2013; Ono et al., 2015; Meng et al., 2022). In 2013, Kawakami et al. (2013) co-cultured mouse early embryonic stem cells (mEES-6) with human salivary gland-derived fibroblasts and found that mEES-6 expressed salivary gland-associated markers, such as amylase, AQP-5, bFGF, and NGF, and possessed similar characteristics to salivary gland cells. After transplantation of salivary gland cells obtained by co-culture into normal submandibular glands of mice, the submandibular glands of mice in the transplanted group were significantly enlarged compared with those in the non-transplanted group without any obvious abnormality in the histological examination, suggesting the formation of functional salivary gland tissues (Kawakami et al., 2013). Meng et al. (2022) found the formation of salivary gland acinar-like and duct-like structures and amylase expression after treatment of iPSCs and IRF6-overexpressing iPSCs using the conditioned medium from human parotid cells. However, this study did not compare the difference in salivary gland cell formation between normal iPSCs and IRF6-overexpressing iPSCs (Meng et al., 2022). In another study, mouse iPSCs were directly transplanted into the submandibular gland of SCID mice to observe the effect of the submandibular gland microenvironment on the differentiation of iPSCs (Ono et al., 2015). It was found that transplanted iPSCs could partially form salivary gland-like tissues and express salivary gland markers such as parotid secretory protein (PSP), amylase, E-cadherin, but the issue of tumorigenicity of iPSCs was not addressed in this study (Ono et al., 2015).

#### 5.1.2 Treatment by transplantation of PSC-derived engineered salivary gland organoids

To date, most tissue regeneration strategies have focused on direct transplantation using specific cell populations, but this is changing with the advent of organoid culture systems (Lancaster and Knoblich, 2014). Salivary gland organoids can be generated from SGSCs/SGPCs or non-saliva gland stem cells (such as dental follicle stem cells or PSCs) embedded in reconstituted extracellular matrix-like materials (such as matrigel, fibrin gel, and collagen gel) (Maimets et al., 2016; Srinivasan et al., 2017; Xu et al., 2017; Tanaka et al., 2018). Due to differences in cell sources and culture methods, these reported salivary gland organoids often do not have consistent cell composition, structure, and gene expression patterns with normal salivary gland tissues. For the purpose of salivary gland organoids to produce saliva and transport it to the oral cavity, salivary gland organoids should have acinar-like structures (to produce saliva), duct-like structures (to transport saliva), myoepithelial cells (to assist saliva secretion) and corresponding neuromodulatory system for saliva secretory (Zhao et al., 2021; Yoon et al., 2022).

The first evidence for transplantation therapy using engineered salivary gland organoids was provided by Tanaka and colleagues (Tanaka et al., 2018). Several small molecules (BMP4, SB-431542, LDN-193189, and FGF2) were firstly applied to induce differentiation of mouse ESCs-derived EBs into oral ectoderm *in vitro*, followed by overexpression of Sox9 and Foxc1 and FGF signaling (FGF7 and FGF10) activating, finally generated salivary gland organoids composed of AQP5^+^ acinar-like cells, CK18^+^ duct-like cells and α-SMA^+^ myoepithelial-like cells were (Tanaka et al., 2018). After orthotopic transplantation of collagen gel-encapsulated salivary gland organoids into mice with defective parotid glands, mouse ESCs-derived salivary gland organoids connect with surrounding tissues and mature (Tanaka et al., 2018). Notably, saliva production was significantly increased after gustatory stimulation of salivary gland organoids transplanted mice with citrate, suggesting that orthotopically transplanted salivary gland organoids reconstitute the afferent and efferent neural pathways required for the regulation of salivary gland function (Tanaka et al., 2018). Another study by Tanaka and colleagues (Tanaka et al., 2022) succeeded in inducing human iPSCs to generate salivary gland organoids with similar gene expression patterns to human embryonic salivary glands after changing partial culture conditions (applying SB431542, FGF7, and FGF10). After co-transplantation of the formed organoid combined with embryonic mouse salivary gland mesenchymal tissue into parotid gland deficient mice *in situ*, further development and maturation of salivary gland organoids were observed, and CD31^+^ endothelial cells and TUBB3^+^ neuronal fibers were detected close to the acinar cells (Tanaka et al., 2022).

In addition to orthotopic transplantation of salivary glands, renal capsule transplantation in nude mice was also used to assess the continued development and function of salivary gland organoids. Sui et al. (2020) combined human submandibular gland stem/progenitor cells to generate salivary gland organoids and embryonic mouse salivary gland mesenchyme heterogeneously transplanted into the renal capsules of nude mice, showing mature salivary gland characteristics, including histological structure (the presence of ducts-like and acinar-like structures with the myoepithelial cells surrounding them) and salivary secretion. In another study from the research group, ESCs were treated with BMP4, retinoic acid, and FGF10 to obtain salivary gland placodes *in vitro* (Zhang et al., 2022). One month after implantation into the renal capsule of nude mice, the salivary gland placodes continued to develop and formed duct-like structures with continuous lumen, but unfortunately, acinar structures were not observed (Zhang et al., 2022).

In addition, SGSCs/SGPSCs or other salivary gland cells combined with different extracellular matrix-like materials to directly generate salivary gland organoids *in vitro* have been reported by several studies (Joraku et al., 2007; Maria et al., 2011; Shin et al., 2016; Srinivasan et al., 2017; Sui et al., 2020). This also provides a new strategy for PSC-derived engineered salivary gland organoid transplantation therapy, that is, PSCs can be differentiated into salivary gland cells first, and then the salivary gland organoids generated from these cells can be used for the treatment of salivary gland diseases (Figure 2). At present, there are no reports of salivary gland organoids for the treatment of SGH disease models, but based on the rapid progress in the research on this field, engineered salivary gland organoids derived from PSCs are expected to play a therapeutic role by replacing hypo-function salivary glands.

### 5.2 Treatment by PSC-derived MSCs

#### 5.2.1 Treatment by direct transplantation of PSC-derived MSCs

MSCs originate from the embryonic mesoderm and are widely found in various tissues, such as the umbilical cord, bone marrow, and adipose, with self-renewal ability and multidirectional differentiation potentials (Pittenger et al., 1999). As mentioned above, the results of several clinical studies have demonstrated the favorable therapeutic effects of stem cell therapy for SS and radiotherapy-induced SGH (Xu et al., 2012; Lynggaard et al., 2022a; Lynggaard et al., 2022b). However, the mechanism of MSCs for the treatment of SGH has not yet been fully clarified. On the one hand, MSCs are able to differentiate directly into salivary gland acinar cells, which holds promise for alleviating SGH by regenerating salivary glands (Lim et al., 2013; Adine et al., 2018; Mona et al., 2020; Yan et al., 2020; Tran et al., 2022). On the other hand, transplantation of MSCs can upregulate the release of anti-inflammatory cytokines and downregulate the release of pro-inflammatory cytokines, while regulating T cells, B cells, dendritic cells, and natural killer cells to perform anti-inflammatory and immune regulation effects (Chihaby et al., 2021). In addition, MSCs can release soluble factors through paracrine effects, which promote cell proliferation and angiogenesis, activate endogenous progenitor cells, and inhibit epithelial cell apoptosis (Lee et al., 2011; Guan et al., 2023).

MSCs are a common source of cells for cell therapy. Still, several factors limit their widespread application in the clinic, such as the need for invasive manipulation for cell isolation, the limited number of isolated cells and expansion capacity, heterogeneity depending on the source and donor (Zhou et al., 2021), Differentiation from homogeneous and well-characterized iPSC cell lines into iMSCs would effectively address these biological and technical limitations. Dozens of studies have reported differentiation strategies for iMSCs, which can be categorized into MSC switch, EBs, specific differentiation, pathway inhibitor, and platelet lysate according to the differentiation procedures (Dupuis and Oltra, 2021). Hai et al. (2018) found that tail vein injection of iMSCs prevented salivary gland lymphocyte infiltration and inhibited further progression of SS in mice through immunomodulatory effects. Unfortunately, this study did not evaluate salivary gland function indicators such as saliva flow rate in experimental animals (Hai et al., 2018). Nevertheless, iMSCs, as an emerging source of stem cells, are expected to play an effective role in the treatment of SGH based on the available animal and clinical evidence.

#### 5.2.2 Treatment by transplantation of iMSC-derived engineered salivary gland organoids

Utilizing the salivary gland differentiation potential of iMSCs to construct engineered salivary gland organoids may become another option for the treatment of SGH. In 2018, an *in vitro* study by Adine et al. (2018) successfully generated innervated secretory salivary gland epithelial organoids, including secretory epithelium, ducts, myoepithelium, and neuronal structures using DPSCs and magnetic 3D bioprinting technology, were able to produce α-amylase in response to FGF10 stimulation. In another study, co-culture of BMSCs with homogenates of decellularized salivary glands extracellular matrix successfully formed cell aggregates expressing salivary gland epithelial cell markers that were morphologically and ultrastructurally similar to submandibular gland tissues (Tran et al., 2022). In further experiments, cell aggregates continued to develop and enlarge after renal capsule transplantation, and generated salivary gland-like organoids (Tran et al., 2022). Whether iMSCs possess salivary gland organoid formation ability similar to that of DPSCs and BMSCs still needs to be verified by more *in vivo* and *in vitro* experiments.

#### 5.2.3 Treatment by transplantation of iMSC-derived paracrine products

Although various MSCs have been extensively explored in tissue engineering or regenerative medicine, the safety of allogeneic MSCs transplantation is still controversial (El-Naseery et al., 2018; Kim et al., 2022) In addition to direct transplantation of MSCs, transplantation of paracrine products derived from MSCs (including EVs and conditioned medium), which are more stable and suitable for long-term storage, has also been reported to help the recovery of SGH without causing pulmonary embolism and the risk of processing tumor formation (Jung et al., 2013; Chansaenroj et al., 2021). EVs are cell-derived membranous structures, enriched with proteins, nucleic acids, and lipids, which can be categorized into exosomes, microvesicles, and apoptotic vesicles based on their diameter (Zhao et al., 2022). Similar to direct stem cell transplantation, the mechanisms of extracellular vesicle-based therapy include immune modulation and tissue regeneration promotion (Harrell et al., 2019; Fujii and Miura, 2022).

Exosomes are currently the most widely studied and used EVs, and have also been tried to be used in the treatment of SGH (Willms et al., 2018). The study by Hu et al. (Hu et al., 2023) found that intravenous injection of exosomes derived from DPSCs could alleviate SS, reduce the level of salivary gland inflammation and increase saliva flow in animal models. SS may cause the death of salivary gland epithelial cells and the decrease of AQP5 expression, which could be reversed by DPSC-derived exosomes through the cAMP/PKA/CREB pathway (Hu et al., 2023). Human UCMSC-derived exosomes could effectively inhibit the over proliferation and apoptosis of CD4^+^ cells and restore the Th17/Treg balance through the autophagy pathway in patients with SS (Ma et al., 2023).

Several studies have reported the improvement of SS in mice treated with intravenous injection of exosomes derived from olfactory ecto- and labial gland-derived mesenchymal stem cells (Li et al., 2021; Rui et al., 2021; Xing et al., 2022). In addition, it was also reported the preventive effect of exosomes derived from tonsil MSCs on submandibular gland dysfunction caused by ovariectomy (Kim et al., 2022). In this study, local injection of tonsillar MSC-derived exosomes in the submandibular gland reversed ovariectomy-induced pro-inflammatory cytokine release, decreased expression of salivary gland secretion-associated proteins (AQP3, AQP5, and α-amylase) (Kim et al., 2022).

Two animal studies found that intravenous injection of iMSC-derived exosomes in the early stage of SS can control the progression of the disease before the onset of salivary gland inflammation (Hai et al., 2018; Zhao et al., 2023). Although the salivary gland function of mice was not further explored in the two studies, based on the injured effect of immune abnormalities in SS on salivary gland function, iMSC-derived exosomes are expected to alleviate SGH caused by SS through immunomodulation. In addition to anti-inflammatory effects, exosomes derived from MSCs have also been reported to promote tissue regeneration, such as regulation of angiogenesis, anti-apoptosis, and improvement of cell aging, through their contained cytokines and microRNA components (Hade et al., 2021). This also makes it possible for iMSC-derived exosomes to be applied to the therapeutic exploration of SGH caused by salivary gland tissue damage.

Conditioned medium of MSCs is another cell-free therapeutic modality that utilizes the paracrine effects of MSCs. Direct support for the application of stem cell conditioned medium is provided by the research of Bi et al. (2007) who found that the use of stem cell conditioned medium can mimic the beneficial effects of stem cell therapy. Matsumura-Kawashima and colleagues (Matsumura-Kawashima et al., 2021) observed a significant reduction in inflammation and a significant increase in the salivary secretion of submandibular glands in mice with SS after intravenously injecting DPSC-derived conditioned medium. However, this therapeutic effect was not found in the BMSC-derived conditioned medium group, which may be related to the fact that the DPSC-derived conditioned medium contains more cytokines that regulate cell proliferation, anti-inflammatory and immunomodulatory effects than the BMSC-derived conditioned medium. Another subsequent study used conditioned medium of human SHEDs for prophylactic treatment of radiotherapy-injured mice (Kano et al., 2023). SHED-derived conditioned medium was found to effectively upregulate the expression of antioxidant genes in mouse salivary glands and human acinar cells damaged by radiotherapy and strongly inhibit the radiotherapy-induced oxidative stress, thereby maintaining the salivary gland function (Kano et al., 2023). There are still few reports of conditioned medium of iMSCs origin for disease treatment. A study in 2021 found that conditioned media derived from iMSCs had a better effect on promoting skin wound healing in mice than that derived from UCMSCs (Liang et al., 2021). Nevertheless, based on the effectiveness of the two kinds of adult MSC-derived conditioned medium for the treatment of SGH and the safety of MSC-derived exosomes, the iMSC-derived conditioned medium has good clinical application potential for SGH.

#### 5.2.4 The potential application of PSC-derived neural crest cell (NCCs) differentiated iMSCs (PNMSCs)

Salivary gland development begins with the migration of neural crest-derived cells and their mediated formation of ectodermal mesenchymal tissue, making NCCs an attractive cell source for the study of salivary gland development and regeneration (Chibly et al., 2022). On the one hand, the paracrine effect of neural crest-derived mesenchyme can help the development and maturation of salivary gland and engineered salivary gland organoids (Patel and Hoffman, 2014; Hosseini et al., 2018; Sui et al., 2020; Zhao et al., 2021). On the other hand, compared with BMSCs, DPSCs derived from neural crest contain more cytokines with tissue regeneration mechanisms (regulating cell proliferation, anti-inflammatory, and immunomodulatory), which can more effectively restore salivary gland function *in vivo* (Matsumura-Kawashima et al., 2021). Therefore, PNMSCs may be a more promising cell source for the treatment of SGH than mesoderm-derived MSCs. Currently, two studies have successfully induced the differentiation of iPSCs and ESCs into PNMSCs *in vitro* (Fukuta et al., 2014; Ouchi et al., 2016; Mitsuzawa et al., 2019). In the future, further research on PNMSCs is still needed to compare their characteristics and the differences in their therapeutic effects on SGH with other MSCs.

### 5.3 Treatment by transplantation of PSC-derived paracrine products

As discussed above, the paracrine products of MSC and iMSCs (EVs and conditioned medium) have strong therapeutic potential for SGH but still need to undergo the differentiation program from PSCs to iMSCs in order to achieve this. At the same time, problems such as genome instability and risk of teratoma formation limit the direct clinical application of PSCs as cell medicines (Suman et al., 2019). Direct treatment with paracrine products of PSCs can address these issues.

Exosomes derived from iPSCs also contains a large number of proteins and microRNA components that promote cell viability, migration and inhibit apoptosis (Sun et al., 2019; Wang, 2021; Bo et al., 2022). Recently, iPSC-derived exosomes have shown therapeutic effects in animal models of various diseases such as cardiovascular diseases, neurological diseases, corneal epithelial defects, and skin wound healing (Oh et al., 2018; Wang, 2021; Pan et al., 2022). In addition, some studies have also reported the therapeutic potentials of iPSC-derived conditioned medium, including inhibiting cardiomyocyte apoptosis, Leydig cell apoptosis, the activation of hypertrophic scar fibroblasts *in vitro* (Ren et al., 2015; Guo et al., 2018; Guo et al., 2019), and reducing retinal light damage, acute lung injury, and acute kidney injury in animal models *in vivo* (Chang et al., 2015; Tarng et al., 2016; Su et al., 2021). Currently, there is no direct evidence of the therapeutic effect of exosomes derived from PSCs on SGH, but their advantages, including safety and simpler isolation procedures, make them an innovative strategy for SGH treatment.

## 6 Conclusion

In summary, SGH is a salivary gland disease caused by several etiologies and is difficult to be completely cured, which seriously affects the quality of life of patients. At present, some clinical and preclinical studies have reported the therapeutic benefits of MSCs and SGSCs/SGPCs and their paracrine products on SGH, but there are still limitations in practical application. As a new source of therapeutic cells, PSCs are expected to address these issues and improve SGH through multiple ways, including the direct transplantation of PSC-derived cells (iMSCs and salivary gland cells), PSC-derived engineered salivary gland organoids, and the paracrine products of PSCs and iMSCs. It is worth noting that although many *in vitro* and animal studies have demonstrated the value of PSCs in the treatment of SGH, no clinical studies on this topic have been reported.

In recent years, the rapid development of three-dimensional bioprinting or Biofabrication has provided new options for the research and application of cell spheroids and organoids and is also one of the future options for regenerative therapy for SGH. Meanwhile, the paracrine products from PSCs and their derived cells may be sufficient to achieve the goal of salivary gland function restoration, which may be expected to replace the common cell transplantation strategies. In addition, the therapeutic hypothesis of PSCs on SGH through different pathways still lacks high-quality clinical evidence to support it, and more rigorously designed clinical/preclinical studies evaluating their safety and efficacy will accelerate the widespread use of PSCs in the treatment of SGH.

In conclusion, this study contributes to the development of next-generation therapies for SGH utilizing PSCs in the future, which is expected to resolve the current clinical dilemma of SGH.

## Abbreviations

SGH, Salivary gland hypofunction; PSCs, Pluripotent stem cells; ESCs, Embryonic stem cells; iPSCs, Induced pluripotent stem cells; EPSCs, Extended pluripotent stem cells; SGSCs/SGPCs, Salivary gland stem/progenitor cells; MSCs, Mesenchymal stem cells; UCMSCs, Umbilical cord MSCs; SS, Sjogren’s syndrome; AT-MSCs, Adipose tissue-derived mesenchymal stem/stromal cells; SGPCs, Salivary gland progenitor cells; iMSCs, iPSC-derived MSCs; BMS, Burning mouth syndrome; USFR, Unstimulated salivary flow rate; SSFR, Stimulated salivary flow rate; SARS-CoV-2, Severe acute respiratory syndrome coronavirus 2; COVID-19, Coronavirus disease 2019; AIDS, Acquired immune deficiency syndrome; HIV, Human immunodeficiency virus; BMSCs, Bone marrow mesenchymal stem cells; DPSCs, Dental pulp stem cells; SHEDs, Exfoliated deciduous tooth stem cells; EBs, Embryoid bodies; mEES-6, Mouse early embryonic stem cells; PSP, Parotid secretory protein; NCCs, Neural crest cell; PNMSCs, PSC-derived neural crest cell differentiated iMSCs.
