# Supplementary material for: Current developments and opportunities of pluripotent stem cells-based therapies for salivary gland hypofunction
Source: Front Cell Dev Biol. 2024 Jan 19;12:1346996. doi: 10.3389/fcell.2024.1346996 (PMC10834761; doi:10.3389/fcell.2024.1346996)
Supplement: Supplementary file 1 [file Table1.docx]

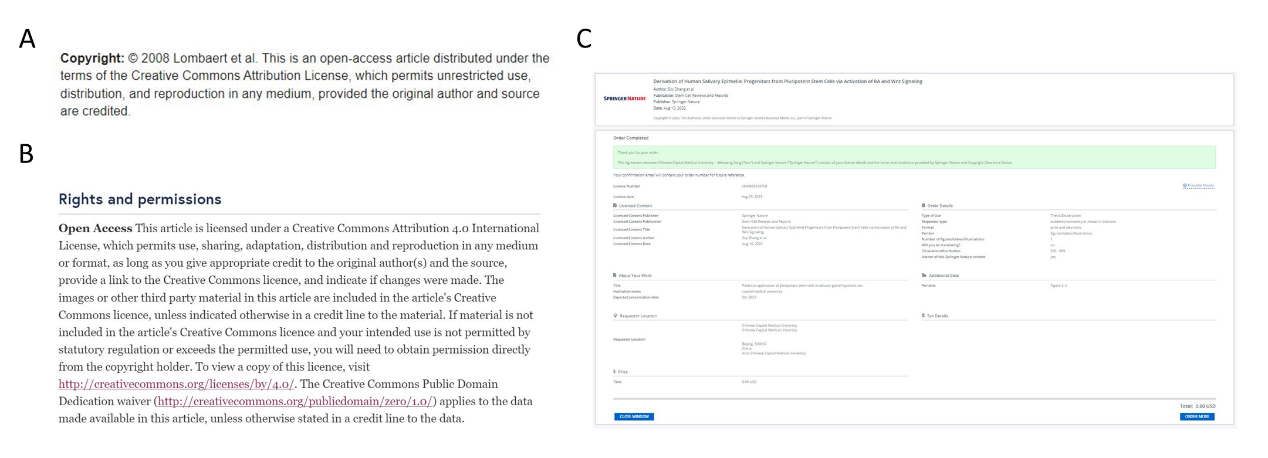


**Supplemental Figure S1.** The obtained permissions for Figure 1. **A.** Figure 1.A was licensed under the terms of the Creative Commons Attribution License. **B.** Figure 1.B was licensed under a Creative Commons Attribution 4.0 International License. **C.** The authors have obtained the permission for figure 1.C using from the Springer Nature.
